# Are All Spatial Reference Frames Egocentric? Reinterpreting Evidence for Allocentric, Object-Centered, or World-Centered Reference Frames

**DOI:** 10.3389/fnhum.2015.00648

**Published:** 2015-12-09

**Authors:** Flavia Filimon

**Affiliations:** ^1^Adaptive Behavior and Cognition, Max Planck Institute for Human DevelopmentBerlin, Germany; ^2^Berlin School of Mind and Brain, Humboldt Universität zu BerlinBerlin, Germany

**Keywords:** allocentric, object-centered, egocentric, spatial reference frames, parietal sensorimotor transformations, place cells, cognitive map, perception and action

## Abstract

The use and neural representation of egocentric spatial reference frames is well-documented. In contrast, whether the brain represents spatial relationships between objects in *allocentric, object-centered*, or *world-centered* coordinates is debated. Here, I review behavioral, neuropsychological, neurophysiological (neuronal recording), and neuroimaging evidence for and against allocentric, object-centered, or world-centered spatial reference frames. Based on theoretical considerations, simulations, and empirical findings from spatial navigation, spatial judgments, and goal-directed movements, I suggest that all spatial representations may in fact be dependent on egocentric reference frames.

## Introduction

Do animals use spatial reference frames that are *independent* of an egocentric viewpoint? In other words, does the brain represent *map-like spatial layouts*, or *spatial locations* of objects and landmarks, in an *allocentric*, or “*other-centered*” *spatial* reference frame, *independent* of the ego's perspective or location? Does the choice of spatial reference frame depend on (passive) perception vs. sensorimotor interactions with the environment, such as target-directed movements or navigation?

It is well-established that neurons in many brain regions, especially parieto-frontal cortex, represent the spatial location of objects in *egocentric spatial reference frames*, centered on various body parts such as the eye (retina), the head, or the hand (Colby, 1998; Hagler et al., 2007; Sereno and Huang, 2014). However, whether the brain also represents spatial locations of external objects relative to other objects in an *allocentric* or *object-centered spatial reference frame*, or constructs an abstract map of such relationships that is independent of the egocentric perspective, is debated (Bennett, 1996; Driver and Pouget, 2000; Wang and Spelke, 2002; Burgess, 2006; Wehner et al., 2006; Rorden et al., 2012; Li et al., 2014).

Here, I review empirical (behavioral, neuropsychological, neurophysiological, and neuroimaging) evidence for and against allocentric vs. egocentric spatial representations. In addition, I discuss theoretical considerations and computational models addressing this distinction.

Based on theoretical considerations and empirical evidence, I suggest that object-centered, allocentric, or world-centered *spatial* representations may be explained via egocentric spatial reference frames. I shall argue that allocentric task effects could alternatively be explained via the following processes:

1. mentally shifting (translating, rotating) an object, thereby lining it up with the egocentric midline (or fovea), such that the object's left (right) and the ego's left (right) are equivalent. Spatial decisions regarding where targets are relative to the object are thus translated into egocentric left/right decisions (*ego-relative remapping*);
2. mental transformations of the ego (e.g., mental rotation or translation of the ego into a new imagined orientation or position, then referencing the location of objects and landmarks to this new, mentally transformed, *egocentric* position);
3. rule-based decision making; for instance, prefrontal top-down control is exerted on a number of brain regions, including on sensorimotor parieto-frontal areas (e.g., top-down inputs from dorsolateral prefrontal cortex to supplementary eye fields or posterior parietal regions such as areas LIP or 7a). Here, rather than using an allocentric *spatial reference frame* to represent *spatial locations*, neurons appear to learn to respond categorically in a *learned, rule-based* fashion, not because of bottom-up construction of an allocentric spatial reference frame based on visual input, but because of categorical signals from prefrontal cortex. This rule-based response only emerges after training, in contrast to, e.g., bottom-up retinotopic representations;
4. object, landmark, or scene recognition, whereby an object, landmark, or scene has been encoded from one or multiple (egocentric) viewpoints (e.g., by medial temporal lobe memory networks). View-dependent object or scene recognition then predominantly activates the ventral, rather than dorsal, visual stream, as well as hippocampal and related structures, depending on the task.

The latter point suggests that landmark or scene recognition via viewpoint-matching is more akin to object recognition than a *spatial* representation of object coordinates and locations relative to an external, environment-based reference frame. As such, the brain might not rely on allocentric spatial reference frames either for spatial judgments in spatial perception, or during navigation, or in sensorimotor transformations for goal-directed movements (e.g., grasping, pointing, or eye movements) toward external objects. Thus, I will argue that neither the way we encode space, nor the way we interact with space, need make use of allocentric spatial reference frames independent of egocentric representations. Object-based *representations* do exist, especially in the ventral visual stream, but are not *spatial* in the sense of referring locations external to the viewer to another external object. Ventral object-centered representations are essentially akin to object recognition, with spatial decisions remaining anchored to a fundamentally egocentric spatial reference frame.

I will commence with some theoretical examples for why it is difficult if not impossible to relate spatial locations (whether left or right, up or down, or simply “the center of”) to external, non-egocentric coordinates. I will then review empirical evidence for different spatial reference frames in navigation, spatial judgments, and goal-directed movements (interactions with spatial targets), as well as computational (simulation) explanations for the effects observed. By attempting to unify a wide range of findings from multiple research areas, this review will necessarily not be fully comprehensive within each domain, but will instead highlight representative studies. Finally, I will conclude with a new suggested categorization of networks contributing to spatial processing, as well as with several predictions made by the egocentric account.

## Theoretical considerations: can spatial representations be independent of the egocentric perspective or position?

Different definitions have been used to define the term “allocentric.” Klatzky (1998), for instance, distinguishes between three “functional modules”: egocentric locational representation, allocentric locational representation, and allocentric heading. Whereas egocentric locational representations reference locations of objects to the observer (ego), allocentric representations reference object locations to space external to the perceiver. For instance, positions could be represented in Cartesian or Polar coordinates with the origin centered on an external reference object (Klatzky, 1998). Allocentric heading, on the other hand, defines the angle between an object's axis of orientation and an external reference direction. Other authors have proposed distinctions between “allocentric” and “object-centered” representations (e.g., Humphreys et al., 2013).

Although different authors have used the terms “allocentric,” “object-centered,” or “world-centered” in many different ways, the majority of the spatial cognition literature has used these terms to refer to representations of spatial relationships between objects or landmarks that do not reference objects' locations to the viewer's body, but to other, external objects (Foley et al., 2015). Here, I shall refer to “allocentric,” “object-centered,” “object-based,” “object-relative,” “world-centered,” or “cognitive map-like” interchangeably, to refer to the representation of the spatial location of an object relative to that of *another* external object, independent of the ego's position or orientation, whether present, imagined, or remembered. This is equivalent to Klatzky's (1998) allocentric locational representation.

In contrast, I shall refer to “egocentric” or “ego-relative” spatial reference frames whenever the observer invokes the position or orientation of the present, remembered, or imagined (e.g., mentally rotated or translated) self, as opposed to an external landmark, to represent the location of external objects.

A spatial reference frame means the receptive field (RF) of a neuron, or the response of the neural population as a whole, is anchored to a particular reference point. For instance, an eye-centered reference frame moves with the eyes (Colby, 1998). A cell preferring stimulation in the left visual field only signals objects when they fall in that cell's RF, which is anchored to the retina. As the eyes move across the visual field, objects' spatial locations change constantly relative to the retina (e.g., an object “left of the eyes” can suddenly be “right of the eyes”). Objects' spatial locations are thus constantly *updated* such that different eye-centered cells, with spatial RFs tiling the visual space, signal the new eye-centered location. An external, “abstract” reference frame, on the other hand, would represent object locations relative to an external reference point, independent of where the observer is (e.g., the location of the microwave relative to the fridge).

For the purpose of this paper it is irrelevant whether neurons with similar reference frames are arranged in a map of space, such as a retinotopic map of space where cells with similar preferences (e.g., “left half of space”) are clustered together. Cells can be *eye-centered* and yet be part of either an orderly *retinotopic map* or a scrambled map of space, with neighboring eye-centered cells having retinal response fields in different locations (Filimon, 2010). I also do not distinguish between reference frames or maps of space represented at the single cell or population level—e.g., the entire population may signal “left of me,” but individual cells' responses may be less clear-cut. The important point addressed here is whether any neural representations, at the single-cell or population level, explicitly signal spatial relationships between objects independent of their spatial location relative to the ego, i.e., whether an explicit object-centered or allocentric spatial representation is formed at whichever computational stage of processing. As defined by Deneve and Pouget (2003), an explicit representation would involve neurons with invariant responses in object-centered coordinates—e.g., the cell should only respond to “left of object,” regardless of where the object is relative to the ego.

Klatzky (1998) also made the distinction between *primitive parameters* conveyed by a spatial representation and *derived parameters* which can be computed from primitives in one or more computational steps. Thus, allocentric location is a primitive parameter in an allocentric locational representation, just like egocentric location is a primitive in the egocentric locational representation (Klatzky, 1998). However, I will review evidence that suggests that allocentric location representations are unlikely to be primitives, but are instead derived from egocentric representations at higher levels of the computational hierarchy, and may not be represented explicitly.

**Figure 1:**
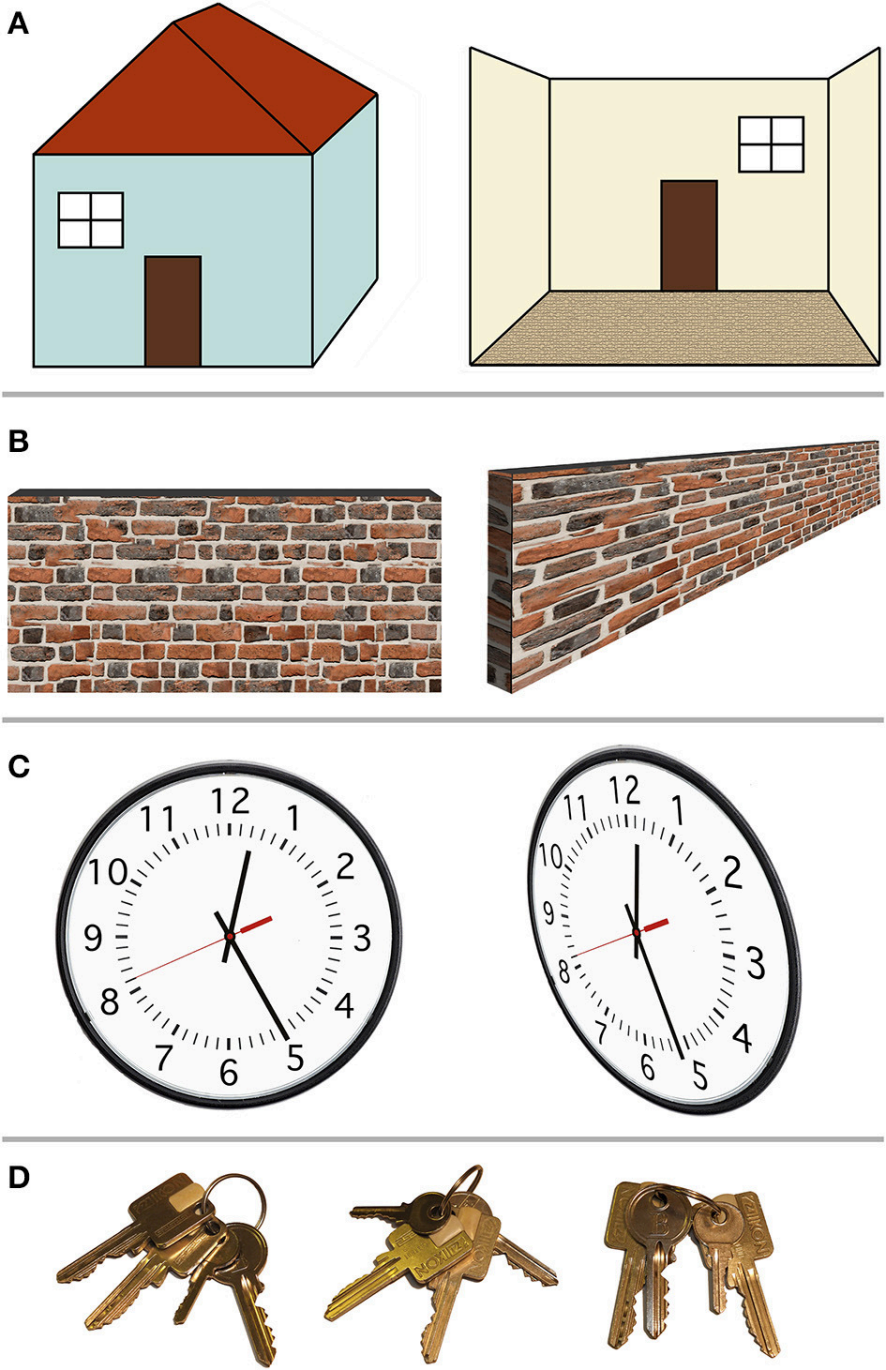
**Example scenarios in which so-called allocentric spatial representations in fact depend on the egocentric viewpoint**. **(A)** Left–right relationships. The window may be defined as “left of the door” (left). However, this only holds when viewing the door and the window from outside the house; when stepping inside the house (right), the window is now “right of the door,” lining up with the egocentric left and right. **(B)** Center-of-object spatial decisions. The center or middle point of a wall (or bar) is easily perceived when the ego is positioned perpendicular to the wall, in front of that center (left). When viewed from the side, however (right), the center point of the wall is harder to determine, because the egocentric perspective distorts the image of the wall on the retina. **(C)** Relative alignment between two objects. The alignment between the minute hand and the individual minute lines (left) suggests the time is 12:25. From a different (egocentric) viewpoint, however, this so-called allocentric spatial relationship shifts, with the new perspective indicating 12:26. **(D)** Proximity (closest to landmark) relationships. Out of two identical-looking square keys, the square key next to the little round key is the one we want. Here, the target square key can be identified independent of the viewer's viewpoint. However, this resembles object recognition with the square and round key forming one unit, followed by rule-based decision making: first identify the little round key, then find the square key closest to it. This seems less like a spatial representation than object recognition.

Figure 1 shows several examples of spatial arrangements that would at first instance appear to be object-based, allocentric spatial relationships. For instance, one could refer to left/right terminology to describe the spatial location of a window relative to a door.

In Figure 1A (left) one could argue that the window is “left of the door” and the door is “right of the window,” regardless of whether the observer is located left of the house (where both the window and the door are on the egocentric right) or to the right of the house (where both objects are on the egocentric left). The fact that the window is “left” of another object, even though it is egocentrically on the right, could be interpreted as an object-centered, ego-independent spatial representation. However, as can be seen in Figure 1A (right), this arrangement is nevertheless dependent on the egocentric viewpoint. Once the observer has walked inside the house, viewing the door and window from the inside, the left–right relationship is reversed: now the window is to the right of the door and the door is to the left of the window. This example demonstrates the ego-dependence of “left” and “right” spatial judgments. The observer merely has to imagine the house aligned with the egocentric center point, such that the house's left (right) and the egocentric left (right) are congruent. Such imagined rotation or imagined translation that transforms the ego's orientation or position relative to an object, or conversely the position of an object relative to the ego, has been called imaginal updating (Klatzky, 1998). Since the definition of left and right depends on the egocentric perspective, this definition of left/right relative to the object (the house or any landmark on it) is not an example of true allocentric or object-centered (ego-independent) spatial representations.

Figure 1B demonstrates another possible way of conceptualizing object-centered spatial representations. Instead of using spatial judgment terms such as “left” and “right,” which appear tied to egocentric perspectives, one could use “center of an object.” Clearly something that is in the center of an object should remain in the center of the object regardless of whether the observer is in front or behind that object. However, as Figure 1B demonstrates, establishing the center point of, e.g., a wall, remains dependent of the egocentric perspective: as soon as the observer is positioned at one end of the object (e.g., at the left end of the wall), the distorted retinal perspective obtained from that (egocentric) location makes it much harder to determine where the center point of the wall is. This may not apply to small objects that can be foveated. However, for small objects (which can be mentally shifted to line up with the fovea), the egocentric left/right and the object's left/right are congruent, and the center point can be estimated based on retinal extent. Alternatively, small objects may be treated as a point in space. As explained above, the critical test for an allocentric representation is independence of object locations from any egocentric perspective, thus relying on abstract spatial relationships *between objects* independent of the observer.

Avoiding “left/right” and “center of” terminology, one might devise a stimulus (Figure 1C) where the relative spatial alignment of two objects is what matters (e.g., the alignment on a clock between the minutes hand and the minute mark corresponding to 25 min). Does the clock indicate 25 min past the hour? As Figure 1C (right) demonstrates, this depends on the egocentric perspective: viewed from the side, the alignment between the minute hand and the twenty-fifth minute mark appears shifted such that one is unsure if the time is 12:25 or 12:26. Thus, even relative spatial alignment between two objects does not appear ego-independent.

Finally, ignoring examples that rely on absolute spatial location (either left or right of center, estimating the center based on distance from the edge, or detecting alignment based on distance between two objects), what about spatial proximity? Figure 1D shows two square keys that appear identical. One of the square keys is located next to a little round key. One can argue that no matter what egocentric perspective one assumes (no matter how the keys are rotated on the key chain), the square key in question will always be closer to the little round key than the other square key. Therefore, this should constitute an allocentric, ego-independent spatial representation. However, rather than involving spatial cognition, this example may rely on *object recognition* followed by *rule-based* reasoning: identify the little key first, then take the square key next to it (regardless of spatial distances or locations). Whereas egocentric spatial selectivity (however malleable) is already present before training, rules need learned. Alternatively, the square key and little key could be encoded holistically as a unit, with one feature activating the entire object configuration in object memory. For instance, in face perception, the spatial location of the nose could be represented relative to the spatial location of the eyes, or the face could be perceived holistically. Holistic object recognition relies on matching entire configurations of features to a stored template. This differs from representing individual features' spatial location relative to other features' spatial location in an allocentric spatial frame, because the spatial relationship between feature A and C should remain unchanged if other parts of the object (features B, D, E, for example) are removed. Logothetis (2000) has argued that not only faces, but even arbitrary objects are processed holistically, as a unit, with neurons responding to particular feature configurations rather than processing individual features.

The examples in Figure 1 primarily pertain to reference frames for *spatial judgments*. However, it could be argued that the main purpose of allocentric spatial frames is navigation and orienting in the environment. Perhaps identifying locations as “north of” or “west of” another object would reveal true allocentric spatial cognition. After all, north remains north regardless of an animal's orientation or location.

**Figure 2:**
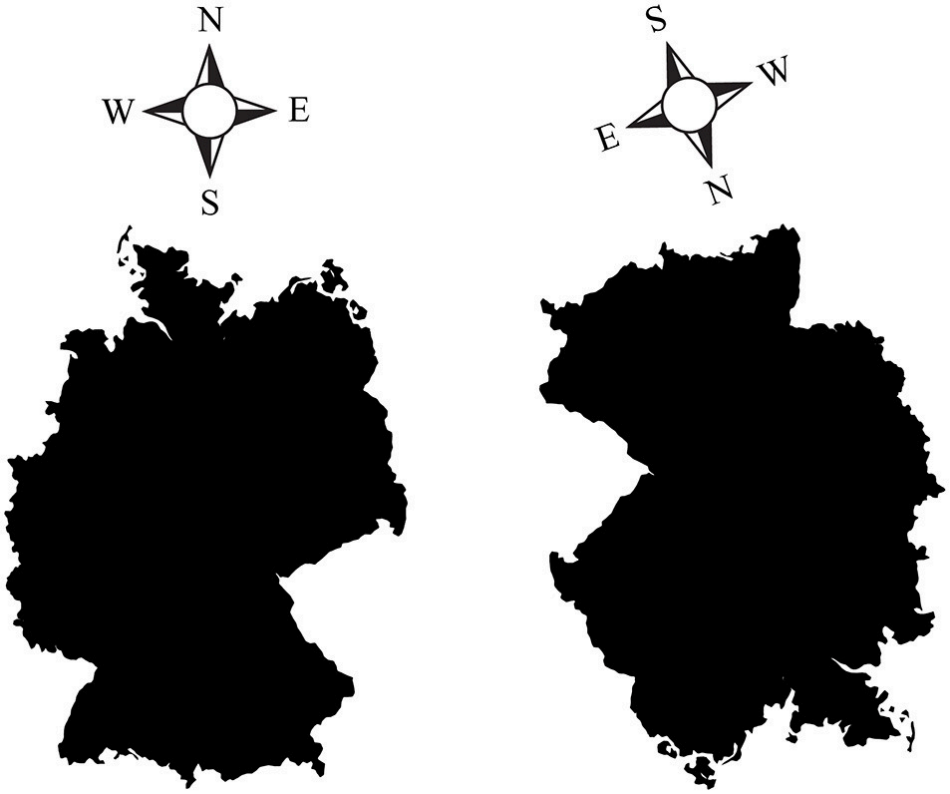
**(Left)** A “right side up” map of Germany, with the four cardinal directions (North, South, West, East) indicated. **(Right)** An upside-down (rotated) map of Germany, with correspondingly rotated cardinal directions. Pointing to Moscow (Russia) is easy with the left map, but harder with the rotated map on the right. Despite the cardinal directions being indicated, it is much harder to orient oneself in the map on the right. This is presumably due to the fact that we tend to mentally line up north, south, west and east with our egocentric coordinates: north is up, south is down, west is left, east is right. As soon as the familiar, egocentric arrangement is disturbed, it takes us longer to mentally rotate what is supposedly an abstract, viewer-independent, hence allocentric map, back up to match our egocentric coordinates.

However, even seemingly external, allocentric, coordinates such as north, south, west, and east may be re-centered on the ego's up, down, left and right coordinates. Figure 2 (left) shows a right-side up map of Germany, with north pointing up. In this orientation, it is easy to figure out, for instance, that Moscow (Russia), located east/north-east relative to Germany, is somewhere slightly *up and to the right* of the image. However, when the map is rotated downward (Figure 2, right), it is much harder to guess where Moscow is, despite the fact that the cardinal directions are still indicated. Why are upside-down maps hard to read? Subjectively, it seems that we perform better when “north” is lined up with the egocentric “up,” and when west and east correspond to the egocentric left and right, because we are then able to rely on our egocentric spatial reference frame to point relative to us. It is likely that most people mentally rotate the map upright to match their egocentric coordinates when making such spatial decisions, rather than relying on an abstract, allocentric map independent of our egocentric coordinates.

Multiple animal species may rely on magnetoreception to orient relative to cardinal directions (Eder et al., 2012; Wu and Dickman, 2012). Note that comparing the ego's heading to an external reference direction is not the same as *allocentric heading* in Klatzky's (1998) terminology, which would involve comparing the axis of orientation of an *external* object and the external reference direction (e.g., “north”). The magnetic field axis appears to be used as an external reference direction to which the *egocentric* axis is compared during navigation. In other words, *the deviation of the ego's axis* from an external axis, not the relationship between one object's axis and another external axis, is signaled. Thus, the question remains: does this magnetic sense allow animals to compute the location of one object relative to another object (e.g., object A is “north” of object B), independent of the animal's orientation, or does it signal “I'm still too far south” or “if I head this way, the destination is ahead?” The latter still entails referencing places in the environment relative to the ego.

The process of aligning oneself with an external axis so that, e.g., north-selective cells receive the strongest stimulation, could be viewed as similar to a primate moving its fovea onto an object in order to get the best (egocentric) viewpoint on it. Aligning one's “magnetic fovea” with the magnetic field's north-south (or east-west, or other) orientation could be viewed as no more allocentric and independent of the ego than aligning one's retinal fovea with a source of visual stimulation in order to get a better (fovea-centered) view of the object, and hence the strongest stimulation. This is also separate from the question of whether *distances* are represented (e.g., “50 miles north of me”), as opposed to local chemical and other sensory cues being used to recognize landmarks upon arrival. Navigating directly toward recognized objects or landmarks does not constitute using an allocentric spatial map (Bennett, 1996).

The question should be not whether an external point or axis can be represented relative to one's own body. This would be equivalent to assuming that “representation of any external point must be allocentric, because that point is, after all, external to the perceiving ego.” Any external point can be represented relative to the ego in egocentric coordinates, thus an external object does not by default imply allocentric processing.

Rather, the question is: are *external* objects represented relative to *other external* object locations, *independent of the egocentric perspective* (whether actual or imagined/remembered)? Evidence for the latter would constitute a true allocentric representation. This is precisely the role hippocampal place cells have been proposed to play in navigation, discussed next.

## Navigation: do place cells, grid cells, and head direction cells form an allocentric map of space?

Upon the discovery of place cells in the rat hippocampus (O'Keefe and Dostrovsky, 1971; O'Keefe and Nadel, 1978), it was suggested that place cells, together with head direction cells and grid cells, form an internal ‘cognitive map’ (Tolman, 1948) of the environment, representing allocentric space (for reviews, see McNaughton et al., 2006; Moser et al., 2008).

**Figure 3:**
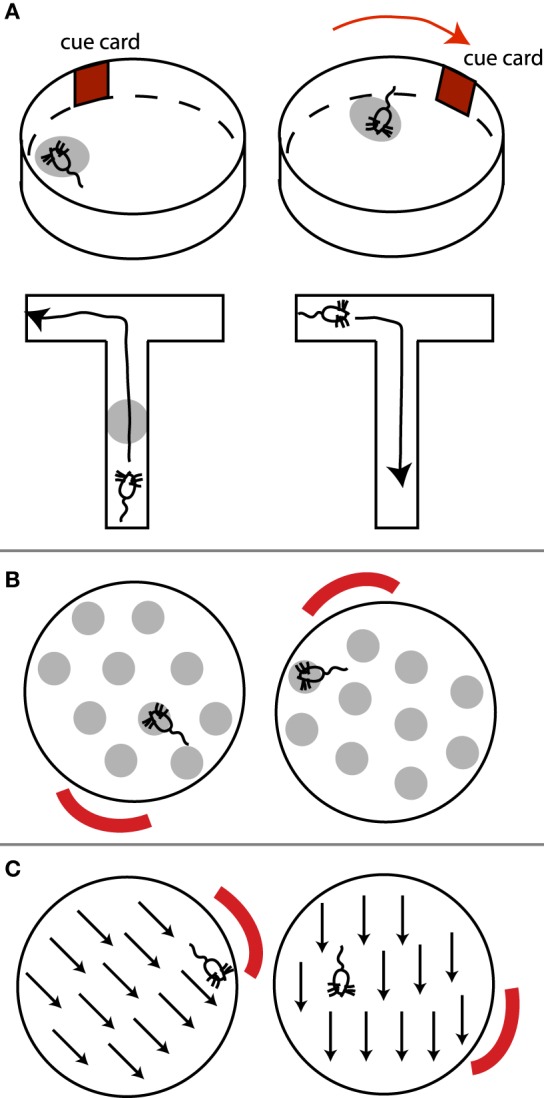
**(A)** Place cells, **(B)** grid cells, and **(C)** head direction cells. **(A)** (top) A place cell's place field (light gray oval) rotates with the rotation of an external cue. Note place field is independent of the rat's orientation within it. **(A)** (bottom) In T-maze environments where routes from one point to the next can be planned, place cells exhibit directional selectivity. This place cell only fires when the rat is moving up the maze, but not when the rat is returning. **(B)** Example grid field rotating with an external cue. The rat's orientation within a grid spot does not matter. **(C)** Preferred direction of a head direction cell rotates with an external cue.

Hippocampal place cells fire at a particular location in the environment (the cell's place field), independent of the rat's orientation inside that place field (Figure 3A, top). “Grid cells,” located in medial entorhinal cortex, display similar spatial tuning, except that each cell has multiple firing fields, effectively forming a periodic array or grid that tiles the environment (Figure 3B; Moser et al., 2008). Similarly, head direction cells (Figure 3C), present in multiple regions including the presubiculum and thalamus, indicate the direction the animal's head is facing, independent of the position or orientation of the animal in the environment (McNaughton et al., 2006). All these cells are anchored to (visual or other sensory) environmental cues (landmarks), and rotate or move their place fields or preferred head direction relative to such external distal cues, if the cues are rotated (Muller and Kubie, 1987; Moser et al., 2008). In other words, these cells appear to signal where the animal *thinks it is located* (or the direction it thinks it is facing). Place fields, grid fields, and head direction signals also persist in the dark, suggesting a reliance on self-motion (path integration) information for maintaining and updating such representations (Moser et al., 2008; e.g., by keeping track of how many steps the animal has taken, or vestibular, head turning signals).

Due to the independence of place and grid fields of the direction from which the animal enters a place or grid field, and hence of the animal's egocentric orientation, it has been suggested that these cells contribute to an allocentric map of the environment (Moser et al., 2008).

However, several pieces of evidence suggest alternative interpretations to an allocentric observer-independent map of space. Although a rat's orientation appears to have no influence on place cells in simple laboratory environments such as high-walled cylinders or open circular platforms, place fields are in fact spatially and directionally selective in environments that require the animal to plan a route between points of special significance, such as in radial mazes where food has been placed (Markus et al., 1995). In such cases, place cells respond at a particular location in the environment only if the animal traverses that location in a particular direction, but not in the other direction (Figure 3A). This contradicts an abstract map-like representation of the environment, since a place on the map should remain the same regardless of how it is traversed. By “abstract map” I mean a “cartographer-like map” independent of the animal's orientation, goals, motivations, memory, or other factors unrelated to the spatial relationship between objects.

In addition, the size of a place field depends on the amount of incoming sensory information. In big brown bats, hippocampal place fields are small immediately after an echolocating call, but rapidly start to diffuse as time passes and echo information decreases (Ulanovsky and Moss, 2011). Moreover, the size of the place field depends on the exploratory mode of the animal: when the bat is scanning the environment from a fixed location using echolocation (akin to a primate saccading around from a fixed position), place fields are more diffuse, and place cells exhibit lower firing rates, than during locomotion through the environment (Ulanovsky and Moss, 2011). The fact that place cells respond differently to the same locations in the environment depending on the animal's behavior, and amount of sensory information received, seems to contradict an abstract map signaling fixed, allocentric, ego-independent relationships between places. After all, the relationship between a door and a window should not change depending on whether the ego is observing this relationship remotely or is passing by. Note that this is unlikely due to a difference in recall: the animal is scanning the landmark in question in both cases, i.e., the landmark has been activated in memory (recalled). What appears to differ is the *egocentric relationship* of the animal relative to the landmark.

Moreover, place fields are over-represented at motivationally salient locations, such as around a hidden platform in a water maze toward which rats are trained to swim (Hollup et al., 2001). This suggests a dependence of the spatial representation on the ego's behavioral goals, rather than a cartographer-like map of the environment.

Place cells are also re-activated during sleep, when the animal dreams about, imagines, or remembers being in a certain place (Pavlides and Winson, 1989). However, this is consistent with the idea that place cells signal *the animal's* current, remembered, or imagined position in the environment relative to some landmark.

Thus, although place cells might appear to encode a cognitive, map-like representation of an environment, place cells might not signal abstract spatial relationships between two places or two landmarks, independent of where the animal is located. Place cells may instead signal *place recognition*, e.g., “I'm by the door,” regardless of whether I have my back to the door or am facing the door. If the door moves (without the animal noticing), a place cell's place field shifts to continue signaling “I am by the door,” even though this is a new geocentric location. Such cells may not indicate “The door is by the window.” In this sense, place cells might act more like object recognition cells than cells that represent spatial relationships between landmarks independent of the observer.

Similarly, while grid cells may map out a regular grid across an environment, with cells responding at fixed, regular intervals as the animal traverses it, the rigid grid-like structure would seem to preclude a flexible spatial representation of one object relative to another object, since no specific object-based relationship is signaled by such an arrangement. Both grid cells and place cells are driven by self-motion cues as the animal keeps track of its changing position (Moser et al., 2008).

Head direction cells signal the animal's heading relative to an external landmark. As described above, however, this signal may compare an egocentric (head) orientation with an external landmark, not the orientation of an external object to a reference landmark.

A recently discovered type of cells, entorhinal border cells, respond along the boundaries of an environment and may form a reference frame for place representations (Solstad et al., 2008). However, such cells do not fire at a distance from a wall or other boundary, but only along the boundary. This may suggest that rather than forming an abstract allocentric reference frame, they signal to the animal “I am near the wall.” Thus, rather than signaling an abstract, allocentric environmental geometry, border cells may similarly represent the ego relative to some landmark, or conversely the landmark relative to the ego, not one landmark relative to another landmark.

Further support for the idea that hippocampal place cells are involved in place recognition in a process more akin to object recognition than spatial cognition comes from recent evidence that human place cells are reactivated during retrieval of objects associated with specific episodic memories (Miller et al., 2013). Participants navigated in a virtual environment, where they were presented with different objects at different locations. At the end of each trial, participants were asked to recall as many of the items as possible, in any order. The authors found that place cells' firing patterns during spontaneous recall of an item were similar to those during exploration of the environment where they had encountered the item. This suggests that recall of objects reactivates their spatial context, but also that place cells encode episodic memories more generally (Miller et al., 2013). Similar to rat place cells (Markus et al., 1995), the majority of human place cells were direction-dependent, only exhibiting place fields when traversed in a particular direction (Miller et al., 2013). This is consistent with an egocentric-dependent viewpoint in scene encoding and recognition, rather than an abstract, allocentric map implemented by place cells.

Finally, it is unknown whether place cells, grid cells, and other types of cells that have been studied in small-scale laboratory environments contribute to navigation in much larger, natural, environments, because it has been impossible to record from such cells in kilometer-sized environments (Geva-Sagiv et al., 2015). In most laboratory experiments, the entire spatial environment can be perceived with little or no movement, meaning that all information needed to calculate the spatial location of different landmarks is available from the animal's current location (Wolbers and Wiener, 2014). This means that in practice, the use of allocentric as opposed to egocentric information may be poorly controlled.

While the functional interpretation of place, grid, head direction, and boundary cells and their contribution to an allocentric map of the environment remains unclear, behavioral studies on animal navigation have also questioned whether animals make use of an allocentric, cognitive map during navigation.

Bennett (1996) has argued that a critical test of a “cognitive map” of space is the ability to take novel shortcuts, instead of following previously experienced routes. According to Bennett, previous evidence for shortcut-taking and putative cognitive maps in insects, birds, rodents, as well as human and non-human primates can be explained more simply either as path integration or recognition of familiar landmarks from a different angle, followed by movement toward them. The animal would thus only need to memorize routes and recognize landmarks to navigate toward them, rather than store a detailed cognitive map of spatial relationships between landmarks. The lack of shortcut-taking ability and hence absence of evidence for a cognitive map is supported by more recent research in a variety of species (Wehner et al., 2006; Grieves and Dudchenko, 2013). Instead, many species appear to rely on view-dependent place recognition, and to match learned viewpoints when approaching landmarks (Wang and Spelke, 2002).

However, when path integration and view-dependent place recognition fail, subjects do appear to be reorienting based on the geometry of a room or based on the “shape of the surface layout” (Wang and Spelke, 2002). Disoriented subjects search for target objects both at the correct corner and geometrically opposite corner of a room—but do not appear to be relying on the spatial configuration between objects (Wang and Spelke, 2002). In other words, not all allocentric information is represented; instead, simpler, geometric layout information is used, which perhaps functions more like object recognition.

In summary, it is unclear if place cells, grid cells, border cells, and head direction cells form the building blocks of an abstract, allocentric map of the environment for navigation, and to what extent these cells are involved in representing the spatial location of an external object relative to another object. Behavioral studies have questioned whether animals actually use an allocentric map for navigation, and whether whichever internal representation is used has the same characteristics as an abstract, cartographic map of the environment (Ekstrom et al., 2014).

## Behavioral studies: spatial reference frames for goal-directed movement

**Figure 4:**
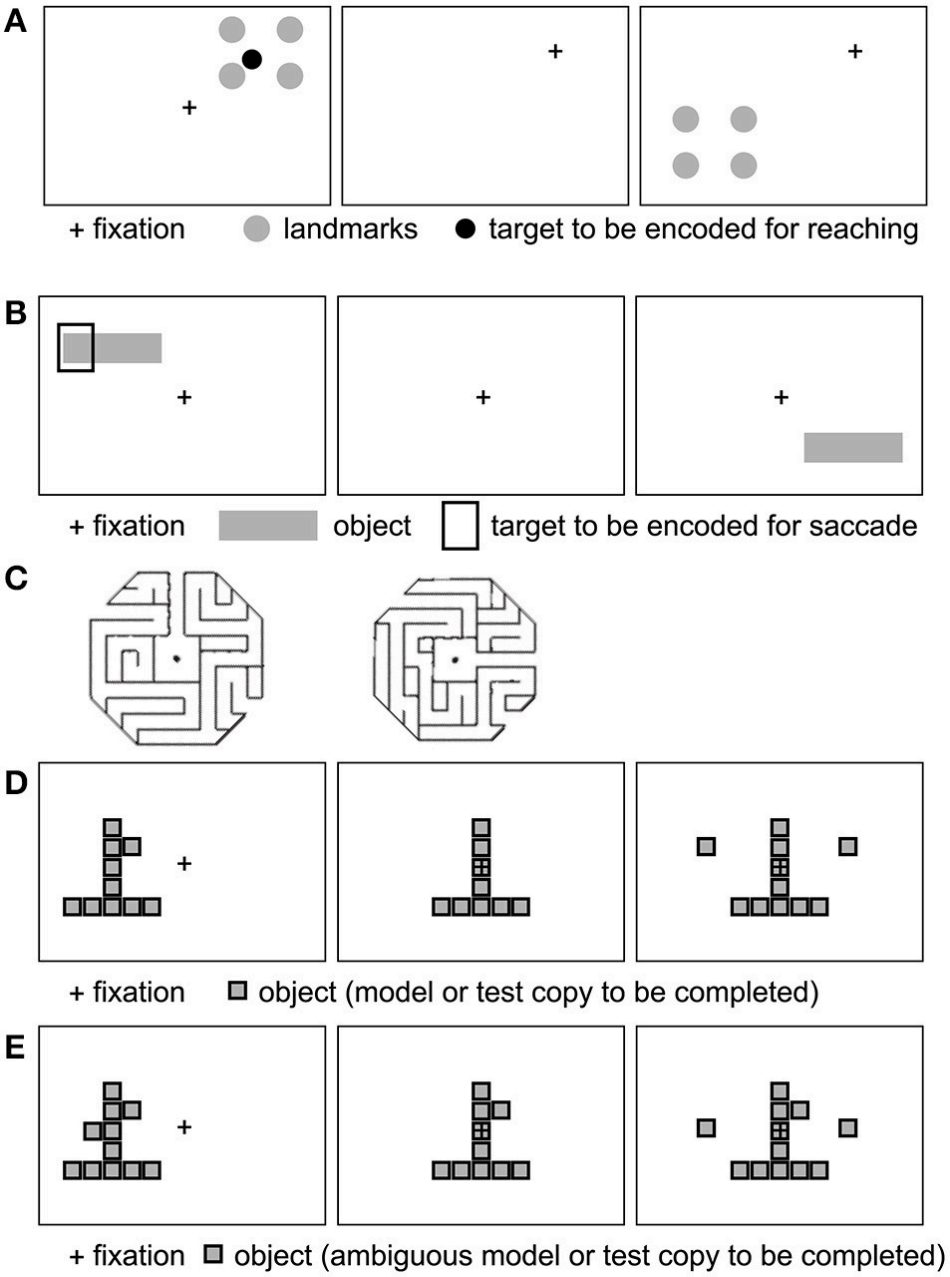
**Example stimuli used to probe allocentric spatial reference frames (see text)**. **(A)** Four landmarks surround an initially displayed reach target. Following a gaze shift (fixation cross moves), the landmarks reappear at a novel location, prompting the subject to point to the remembered target relative to the landmarks. Example based on Chen et al. (2011). **(B)** A target is displayed relative to a horizontal bar. After a delay, the bar reappears without the target. The monkey saccades to the bar-relative location of the target. Inspired by Olson (2003). **(C)** Example maze stimuli to test for maze solving. Adapted from Crowe et al. (2004), with permission. **(D, E)** Example object construction tasks. Panel **(D)** shows a model, followed by the removal of a critical element defined in relation to the model object. Following a delay, the monkey selects the missing piece to complete the object. **(E)** Ambiguous model object: the monkey does not know which of the two knobs (left and right squares) will be removed. Adapted from Chafee et al. (2005) (see text), with permission.

Several behavioral studies have investigated which spatial reference frames are used in goal-directed actions, such as (delayed or immediate) pointing, reaching, grasping, or saccades to (visual or remembered) targets (for reviews, see Battaglia-Mayer et al., 2003; Crawford et al., 2011). Many such studies have investigated spatial reference frames in the context of spatial updating (Colby, 1998; Crawford et al., 2011), where a spatial target is briefly presented, followed by a change in gaze direction before the reach (or saccade) to the remembered location of the target. Saccade or reach endpoint errors and other metrics can then be investigated in the context of landmarks being present vs. absent at the moment of target presentation (Figure 4).

Substantial evidence exists for gaze-centered (egocentric) updating of reach targets following an intervening saccade, for both immediate and delayed movements (Henriques et al., 1998; Medendorp and Crawford, 2002; Thompson and Henriques, 2008; Rogers et al., 2009; Selen and Medendorp, 2011). These studies suggest that the spatial location of a visual target is maintained in an eye-centered reference frame (i.e., as the retinal distance between the current gaze direction or fixation point, and the remembered target location), and is updated across eye movements. While some evidence suggests that gaze-centered updating persists even after long delays (Fiehler et al., 2011), others have suggested that allocentric spatial representations are used when movements are delayed (Westwood and Goodale, 2003).

Several studies have demonstrated more accurate reaching in the presence of landmarks following gaze shifts (e.g., Byrne et al., 2010), and that integration of egocentric and allocentric or landmark information may depend on the stability of visual cues; i.e., the weight assigned to landmarks depends on whether the landmark is moving around (Byrne and Crawford, 2010). Note that the presence of a landmark should not automatically be assumed to involve allocentric (object-centered) reference frames. Both the landmark and the target could be represented relative to the ego. However, can behavioral differences between memory-guided reaches with and without landmarks be explained without relying on the assumption that an allocentric spatial reference frame is used? What accounts for the observed behavioral effects? I will describe two representative experiments in detail to illustrate how *egocentrically*-encoded landmarks could contribute to such differences.

In a study by Schütz et al. (2013), subjects reached to remembered target locations after intervening saccades, either in the presence or absence of visual landmarks. Subjects foveated a briefly displayed target, and continued fixating its location after its disappearance. After a delay of 0, 8, or 12 s subjects then saccaded to a new fixation cross which appeared at various visual eccentricities. Following the gaze shift, the fixation cross also disappeared and subjects reached to the remembered target location in complete darkness. In the allocentric condition, two light tubes were present left and right of the screen, respectively. Pointing errors varied systematically with gaze shift, e.g., when fixating to the left, subjects overshot the remembered target location in the opposite direction, in both the visual landmark and the no-landmark condition. This is consistent with previous evidence that reaching is carried out in eye-centered (hence, egocentric) coordinates (Henriques et al., 1998). Moreover, the different delays led to similar reach endpoint errors, i.e., the effect of the (egocentric) gaze shift remained the same regardless of a delay or not. This suggests that both immediate and delayed reaches rely on gaze-dependent (egocentric) spatial representations.

In addition to varying with gaze shift (an egocentric influence), however, endpoint errors were reduced in the landmark condition. One possible interpretation of this landmark influence is that egocentric and allocentric spatial representations are combined (Schütz et al., 2013). While it is possible that reach targets are represented relative to both landmarks and gaze position, an entirely egocentric explanation cannot be ruled out. For instance, both the initial target and the landmarks could be represented in gaze-centered coordinates. In the no-landmark condition, the target disappears before the fixation cross reappears at a novel location, with the subject sitting in complete darkness during the variable delay. When the novel fixation cross appears, the egocentric estimate of how far the eyes have moved relative to the remembered target (the retinal distance) is less precise. Even in the 0 s delay condition, the target still disappears before the new fixation cross appears, i.e., the new fixation location and the target are never simultaneously displayed, which may lead to a less precise calculation of the saccade vector from (former) target location to (novel) fixation cross location. Previous research (Chen et al., 2011) has shown egocentric information decays gradually, with decay commencing as soon as the target disappears (Westwood and Goodale, 2003). In the absence of external visual landmarks, these factors could thus contribute to a less accurate estimation of how far the eyes have moved away from the initial target location, or greater uncertainty regarding gaze position relative to the former target location (in retinal coordinates), when the reach is initiated. In contrast, in the allocentric landmark condition the landmarks are present throughout the trial, which can lead to a more accurate retinal (egocentric) estimate of how far the eyes have moved. Subjects can represent both the target and the landmarks relative to their gaze when initially viewing the target, and update this eye-centered representation after the saccade. For instance, the left landmark may be at −10° of visual angle relative to the target in the beginning, and at −5° after the saccade to the new fixation cross, when the reach target has disappeared. The gaze shift vector (in eye-centered coordinates) will thus be estimated more accurately, and can be subtracted from the previous eye-centered position of the hand, to more accurately lead the hand to the remembered target position (in eye-centered coordinates, e.g., Medendorp and Crawford, 2002).

Thus, although the combination of allocentric and egocentric cues remains a possibility, the reduced endpoint reach error in the landmark condition could be explained in terms of less accurate egocentric updating. This explanation is more parsimonious, as it involves a single (egocentric) spatial reference frame. To tease apart these competing accounts, the egocentric account makes a testable prediction: if the new fixation cross were to appear before the target is extinguished, there should be reduced uncertainty regarding how far the eyes have moved, even in the absence of landmarks, and hence reduced endpoint errors, similar to the landmark condition. Future experiments could address this prediction. A second prediction could be tested to tease apart allocentric vs. egocentric influences: the two light tubes (landmarks) could be briefly turned off at the same time as the target, during the saccade to a new fixation cross. The landmarks could reappear just before or at the time of the reach. The prediction is that a disruption in egocentric updating of how far the eyes have moved will lead to greater reach error, even when the landmarks reappear later. This would support an egocentric explanation of the landmark effect.

In another study, Chen et al. (2011) compared the rate of memory decay for egocentric and allocentric reach targets, using delayed reaching to remembered target locations following intervening saccades.

In the egocentric condition, a target appeared in the periphery relative to the fixation cross. After the target disappeared, subjects shifted their gaze to a new fixation location. Following a variable (short, medium, or long) delay, the fixation cross disappeared, and subjects reached to the remembered (and egocentrically remapped) location of the reach target.

In the allocentric condition (Figure 4A), the target was surrounded by four landmarks. These landmarks reappeared at a different location following the short, medium or long delay after the gaze shift, and subjects reached to the remembered (and remapped) target location, relative to the landmarks.

In a similar third condition, the allo-to-ego conversion condition, the four landmarks reappeared at the new location both before and after the variable delay.

The authors found that in the egocentric and allo-to-ego conversion condition, reaching variance (endpoint error, reduced precision) increased from short to medium delays, whereas reaching variance remained constant across delays in the allocentric condition. Similarly, reaction times in the egocentric and allo-to-ego conditions were longer at short delays compared to longer delays, whereas reaction times did not vary according to delay in the allocentric condition. The authors concluded that egocentric representations of target locations decay faster than allocentric representations. It was also suggested that allocentric information is converted to an egocentric representation at the first possible opportunity (Chen et al., 2011). Thus, the allocentric landmarks appearing both before and after the delay in the allo-to-ego condition could be used to infer the location of the target in egocentric coordinates before the delay (an allo-to-ego conversion at the first opportunity), and this egocentric information decays with increasing delays. This interpretation could explain the increase in endpoint errors across delays in the egocentric and the allo-to-ego conditions, and the absence of a modulation by delay in the allocentric condition (when landmarks only appear after delays).

Can these behavioral differences between egocentric and allocentric conditions be explained using a purely egocentric reference frame? It is possible that both the target and the surrounding landmarks were represented in egocentric coordinates, and were mentally shifted to center on the fovea (i.e., the center of mass of the square in Figure 4A would line up with the fixation point). As such, a target closer to e.g., the bottom left landmark would also be in the egocentric lower left relative to the fovea. When the landmarks reappeared at a new egocentric location, the new target location could be remapped in egocentric coordinates based on shifting the entire structure (landmarks plus retinocentrically remapped/remembered target) to the new retinal location. Alternatively, even without mentally shifting the landmarks to imagine them around the fixation point, retinal distance vectors can be computed from the fixation point to both the landmark nearest the target (“vector x”) and to the target (“vector y”). The difference between vectors x and y can be stored as a retinal vector (“z”). When the landmark reappears at a different location in the visual field, the retinal vector to its (egocentric) coordinates is calculated, and the difference vector z can be added to infer the new target location in egocentric, rather than allocentric, coordinates. Egocentric remapping of targets has been demonstrated in multiple brain regions (Colby, 1998).

Why then were there differences between egocentric and allocentric reach accuracies and reaction times? Unlike in the egocentric condition, the allocentric landmarks reappear after the delay, just before movement onset, thereby facilitating remapping of the remembered target in egocentric coordinates just before movement onset. Since the landmarks are displayed just before movement onset in each of the three delay conditions, with the delays preceding, not following, the reappearance of the landmarks at the new location, the (egocentrically) remapped location does not get a chance to decay before movement onset. This could explain the shorter and constant reaction times in the landmark condition compared to the egocentric condition. In contrast, in the egocentric condition no new cues are presented after the intervening saccade and variable delay. The longer the delay, the greater the egocentric information decay, consistent with the authors' interpretation (Chen et al., 2011).

What about the allo-to-ego condition, which resembled the egocentric condition in terms of an increase in reach errors across delays? In the allo-to-ego condition, the amount of time the landmarks are displayed at the new location is halved: instead of reappearing for 1.5 s after the delay, they appear for only 0.75 s before and 0.75 s after the variable delay. This shorter presentation time may have led subjects to rely on the first reappearance of the landmarks to update both landmarks and the target in egocentric coordinates, as suggested by the authors. Since the variable delay follows the first reappearance of landmarks, egocentric information decays just like in the no-landmark, egocentric, condition.

In summary, although it is possible that a fundamental difference exists between egocentric spatial representations, thought to decay rapidly across delays, and allocentric spatial representations, which are thought to be more stable and decay less rapidly, these results are equally compatible with an egocentric remapping of all targets, whether surrounded by landmarks or not, accompanied by an egocentric decay in all cases where the remapped information precedes a variable delay. This and similar studies therefore do not necessarily demonstrate the existence of allocentric spatial representations.

Behavioral studies have also investigated visual illusions such as the Müller–Lyer illusion, in which a line segment is flanked by either pointed arrow heads or arrow tails. Subjects perceive identical-length segments with arrow tails as longer than those with arrow heads, which could be interpreted as evidence of allocentric encoding of object features relative to each other. However, Howe and Purves (2005) have shown that this illusion can be explained by natural image statistics where the physical sources giving rise to a 2D retinal image of a line segment with arrow heads tend to belong to the same plane (object, or surface area), whereas physical sources for arrow tails are less likely to come from the same plane. The illusion could thus arise from a probabilistic interpretation of 2D retinal projections of the real world—and would not require allocentric spatial encoding of individual features. A review of 33 studies of pointing to Müller–Lyer stimuli showed that visually-guided pointing (rather than from memory) is typically not subject to the Müller–Lyer illusion, suggesting that this illusion is mediated by the ventral rather than dorsal visual stream (Bruno et al., 2008).

Other studies have investigated pointing accuracy to surrounding objects after subjects were disoriented through self-rotation, with objects hidden from view (Wang and Spelke, 2000, 2002). In such experiments subjects show increased configuration pointing errors, i.e., a deterioration in the internal representation of the angular relationship between targets (e.g., where the TV is relative to the table). This has been interpreted as a disruption to dynamic egocentric updating of target locations (relative to the current ego location), even after controlling for vestibular stimulation, re-orientation via an external light, and other factors, contradicting an enduring cognitive map of allocentric spatial relations between objects independent of the observer (Wang and Spelke, 2000).

Conversely, other studies have shown that disorientation leads to much lower error in “judgments of relative direction” (JRD tasks), where, rather than pointing from the current ego location to objects' locations, subjects imagine themselves by an object and point to another object from that imagined location (e.g., imagining the ego by the door and pointing toward the TV from that location; Burgess, 2006; Waller and Hodgson, 2006; Ekstrom et al., 2014).

However, it is unclear whether higher performance in the JRD task necessarily means subjects rely on stored allocentric representations of object locations relative to each other. The JRD task may simply involve accessing stored egocentric viewpoints, mentally rotating (shifting) the ego to one of the objects, and making an egocentric decision as to where objects are—relative to the ego. In Waller and Hodgson's study (Waller and Hodgson, 2006), for example, participants walked past each of the objects to be encoded, thereby presumably obtaining multiple egocentric viewpoints on the scene layout. Disorientation does not affect JRDs compared to pointing from the current ego orientation, because JRDs rely on stored *egocentric* viewpoints, whereas orientation-dependent pointing requires re-establishing ego-relative object locations anew. Behavioral differences or effects between two experimental conditions thus do not necessarily demonstrate that allocentric vs. egocentric spatial reference frames are used. The two tasks can be viewed as different egocentric tasks, with differences due different egocentric mechanisms being activated (mental rotation of the ego and recall of egocentric viewpoints vs. remapping current target locations relative to the ego following disorientation). Such mental rotations are supported by evidence that recognition times of arrays of objects displayed on a circular table, when rotated to various degrees, increase linearly with the angle of rotation away from the original display (Wang and Spelke, 2002).

If allocentric tasks can be solved by mentally rotating or shifting either the ego or a display of landmarks back to an egocentric (perhaps retinal) center, what if only subsets of objects are shifted in a scene—could reach errors reveal whether subjects encode targets relative to objects rather than the ego? Fiehler et al. (2014) found that the greater the number of objects shifted, the greater the deviation of reach endpoints in the direction of object shifts. While this suggests a plausible allocentric mechanism whereby target locations are encoded relative to other objects, rather than relative to the ego, this could depend on whether an egocentric reference point is provided during encoding of object (target) locations. If a retinal reference point is missing (no fixation cross provided during encoding), subjects may not notice shifts in clusters of objects and still rely on view-dependent (partial) scene recognition, with reaching performed relative to a presumed egocentric reference point that could not be accurately established during encoding. Shifting single large or single smaller local objects had no effect on reach endpoint errors (Fiehler et al., 2014). Similar view-dependent local scene encoding or retinal visual distance calculations can account for other studies in which combined egocentric and allocentric influences were examined (Byrne and Henriques, 2012; Camors et al., 2015).

## Neuropsychology: object-centered spatial neglect?

A number of neuropsychological studies of hemineglect patients have identified seemingly dissociable egocentric vs. object-centered (or allocentric) neglect symptoms, as well as dissociable brain damage sites (for reviews and critiques, see Olson, 2003; Rorden et al., 2012; Yue et al., 2012; Humphreys et al., 2013; Li et al., 2014).

Following damage to (predominantly) the right hemisphere, patients exhibit unawareness of the contralateral (egocentric left) side of space (Humphreys et al., 2013). In addition to egocentrically-defined hemineglect, some patients ignore the left half of an object or of objects, even if presented in their intact (egocentrically right) hemifield, or even if rotated such that the left half of the object falls on the (intact) right visual field (e.g., Caramazza and Hillis, 1990; Driver and Halligan, 1991; Behrmann and Moscovitch, 1994; Behrmann and Tipper, 1994; for review, see Humphreys et al., 2013). The fact that the left half of an object is neglected even when rotated and presented in the egocentric right half of space has been interpreted as evidence for object-centered spatial representations.

However, alternative explanations have been proposed for this pattern of object-based hemineglect. For instance, rotated objects presented in non-canonical orientations may be mentally rotated back upright to match an egocentric, canonical (mental) representation of the object, the left half of which is then ignored (Buxbaum et al., 1996; Humphreys et al., 2013).

Similarly, computational models suggest that a decreasing attentional gradient from (the egocentric) right to left could lead to the left half of any item anywhere in the visual field being less salient and therefore more likely to be ignored (Driver and Pouget, 2000; Pouget and Sejnowski, 2001). Models relying on such “relative egocentric neglect” (Driver and Pouget, 2000; Pouget and Sejnowski, 2001) have successfully modeled what appears to be object-centered neglect (Pouget and Sejnowski, 1997, 2001; Mozer, 1999, 2002).

Such a lesion-induced (*egocentric*) gradient of salience, which could affect either the stored representation of an object or the allocation of attention to this representation, is supported by evidence that the severity of allocentric neglect is modulated by egocentric position, with milder allocentric deficits at more ipsilesional egocentric positions (Niemeier and Karnath, 2002; Karnath et al., 2011). The field of view across which such a gradient in salience is exhibited may be flexibly adjusted (similar to a zoom lens; Niemeier and Karnath, 2002; Karnath et al., 2011; Rorden et al., 2012). For instance, exploratory eye movement patterns in neglect patients did not differ between egocentric and allocentric neglect, but rather differed according to the task goal and strategies, with the same item either detected or neglected depending on the task (Karnath and Niemeier, 2002).

However, double dissociations between egocentric and allocentric neglect have been reported, together with apparent double-dissociations in lesion sites (Humphreys and Heinke, 1998; Humphreys et al., 2013). Egocentric neglect tends to be associated with more anterior sites in supramarginal gyrus and superior temporal cortex, whereas allocentric neglect tends to correlate with more posterior injuries such as to the angular gyrus (Medina et al., 2009; Chechlacz et al., 2010; Verdon et al., 2010).

In contrast, several recent studies have reported that allocentric neglect co-occurs with egocentric neglect, and that the lesion sites overlap (Rorden et al., 2012; Yue et al., 2012; Li et al., 2014). Rorden et al. argue that previous studies have used vague or categorical criteria in classifying patients with allocentric vs. egocentric neglect, leading to an apparent double dissociation between deficits. (For instance, a patient with both egocentric and allocentric deficits would be categorized as allocentric-only, leading to an apparent double-dissociation). To identify whether egocentric and object-centered neglect are dissociable, Rorden et al. used a “defect detection” task in which right-hemisphere stroke patients had to separately circle intact circles and triangles as well as circles and triangles with a “defect” (e.g., a gap in the left half of a circle). Unlike previous studies, which had coded allocentric and egocentric neglect in a categorical, dichotomous manner, thereby ignoring the varying severity of deficits, Rorden et al. used a continuous measure. Allocentric neglect scores were calculated based on the number of correctly detected items with defects as well as intact items correctly marked, on both the contralesional and ipsilesional side. In addition, they also used a center of cancelation task to calculate egocentric neglect scores based on how many targets (e.g., the letter A) were identified in a cluttered field of letters, weighted according to their position from left to right.

Confirming previous findings by Yue et al. (2012), Rorden et al. (2012) found that allocentric deficits were always observed in conjunction with egocentric deficits, with no pure cases of allocentric neglect. In contrast, egocentric neglect did occur on its own. The allocentric neglect score was strongly correlated with patients' egocentric neglect score, and substantial allocentric neglect was only present with substantial egocentric neglect, suggesting that allocentric neglect is a function of severe egocentric neglect.

Moreover, the regions of brain damage associated with egocentric and allocentric neglect strongly overlapped. Rorden et al. (2012) suggest that previous findings of an association between posterior temporo-parietal lesions with allocentric neglect, and superior and middle temporal lesions with egocentric neglect, may in fact result from the same mechanism, namely the extent to which the middle cerebral artery territory is affected by stroke. According to this account, allocentric deficits may be subclinical in milder forms of neglect, which are associated with damage restricted to the central aspect of the middle cerebral artery territory, thus producing what appears to be purely egocentric neglect. In contrast, more severe forms of neglect, comprising both egocentric and allocentric deficits, are due to damage to a larger extent of middle cerebral artery territory, including more posterior regions typically associated with allocentric neglect.

The fact that patterns of object-centered neglect can be explained in terms of an egocentric gradient in salience, as well as recent evidence of a lack of double-dissociation between egocentric and allocentric neglect symptoms and lesion sites, argue against independent egocentric and allocentric spatial representations, and support a single (egocentric) mechanism.

Other neuropsychological investigations have focused on lesions to the ventral visual stream. For instance, patient D.F. shows impairment in (conscious) visual shape perception, but accurate visuomotor performance (such as correct grip aperture) in actions directed to different object shapes (Goodale and Milner, 1992; Goodale and Humphrey, 1998). This has been interpreted as evidence for separate vision for perception (ventral) and vision for action (dorsal) streams (Goodale and Milner, 1992). Schenk (2006) has questioned whether D.F.'s impairment is perceptual, rather than allocentric. In Schenk (2006), D.F. was impaired on a visuomotor task that involved proprioceptively-guided pointing to the right or left of the current hand position by a similar amount as displayed visually between a visual cross and visual target. As suggested by Milner and Goodale (2008), however, the impairment could have been due to the task requiring D.F. to make a perceptual judgment (visual estimate) of the distance between the visual stimuli, before being able to translate that visual distance into a visuomotor plan to a different location. Moreover, this estimate could happen via a “perspectival” (egocentric viewpoint-dependent) mechanism (Foley et al., 2015), rather than an allocentric mechanism. The latter interpretation would thus suggest the ventral visual stream is involved in perceptual (e.g., visual size) estimates underlying shape perception, not necessarily allocentric spatial cognition.

Foley et al. (2015) have argued that whereas the dorsal visual stream uses effector-based egocentric spatial representations, the ventral visual stream may use a perspectival egocentric representation of scenes or objects. Note that this perspectival account is compatible with holistic configural scene or object processing (Logothetis, 2000). Moreover, according to Foley et al., the purpose of ventral visual stream computations is object recognition, attaching emotional or reward value to such a representation, or habitual learning (i.e., what to do with such an object, regardless of the current egocentric perspective on it).

These proposed processes are consistent with the findings presented in the present review, and are compatible with an egocentric account of spatial processing.

## Neurophysiology: evidence for object-based spatial representations, or rule-based decisions?

Neurophysiological studies have shown that multiple egocentric (e.g., hand-centered and eye-centered) representations of the same target can co-exist in parallel or change fluidly during sensorimotor transformations (Battaglia-Mayer et al., 2003). In fact, many neurons exhibit hybrid (e.g., both eye and hand-centered) reference frames (Avillac et al., 2005; Mullette-Gillman et al., 2009). Here I examine whether single-unit neurophysiology evidence supports the representation of an allocentric reference frame at the neuronal level at any point in the sensorimotor transformation. A number of single-unit recording studies have reported object-centered spatial representations in both prefrontal and posterior parietal cortex. In a series of studies, Olson and colleagues (Olson and Gettner, 1995, 1999; Olson and Tremblay, 2000; Tremblay et al., 2002; for review, see Olson, 2003) reported object-centered spatial selectivity in macaque supplementary eye field (SEF) neurons during saccade planning. A typical task (Figure 4B) involves first presenting a horizontal bar with a cue left or right on the bar, at various retinal locations, while the monkey is fixating centrally. Following a variable-duration delay, the horizontal bar is presented at another location in the visual field. After a second variable-length delay, the fixation point disappears and the monkey executes a saccade to the remembered target location relative to the object, i.e., left or right on the bar, regardless of whether the bar is now in the left or right visual field. Interestingly, many SEF neurons show differential activity during the post-cue delay prior to object-left vs. object-right saccades, even though the monkey does not yet know the direction of the *physical* saccade. In other words, while the monkey is holding the object-centered location in working memory, after the bar and cue disappear, but before the new horizontal bar appears, SEF cells selectively signal object-right vs. object-left locations, suggesting object-centered spatial selectivity. This effect is also obtained if color cues or discontinuous objects/cues (e.g., left vs. right of two dots) are used to instruct left vs. right saccades relative to the object (Olson, 2003).

While these results are consistent with object-centered spatial representations in SEF, several additional findings allow for an alternative interpretation. For instance, the neurons that prefer the bar-right condition are predominantly in the left hemisphere, while bar-left neurons predominate in the right hemisphere (Olson, 2003), consistent with an egocentric contralateral representation of each half of space. The fact that neurons selective for *object* coordinates are arranged according to *egocentric* space in the brain could suggest a *recentering* of the *mental representation of the object* during the delay, such that the left half of the object falls in the (egocentrically) left visual field and the right half of the object in the (egocentrically) right visual field.

The idea that a (re-)centered mental representation is driving these responses is also supported by other characteristics of SEF neurons' responses: the object-centered spatial selectivity emerges during the post-cue delay, even when the new target bar isn't visible yet, i.e., before a new object-relative target position can be calculated (e.g., Figures 1, 4, in Olson, 2003).

Interestingly, color cues take longer (200 ms) than spatial configuration cues to evoke object-centered activity, suggesting a top-down, rule-based decision process, perhaps coming from other prefrontal regions such as dorsolateral prefrontal cortex (DLPFC). SEF neurons can also learn to respond to color instructions even if the color cue that signals an object-left rule appears at the right of the object (dot array; Olson, 2003). In such cases, the neuron indicates both the object-relative location of the cue (i.e., if the neuron prefers left on the object, yet the cue signaling a future left-object saccade appears on the right, the neuron responds weakly to the cue) and the object-relative location of the target (i.e., if the target then appears on the left in a left-object preferring neuron, a strong response is obtained; Olson, 2003). This pattern has been interpreted as object-centered spatial selectivity, and that the target could not be selected by an *object-centered rule* (since the cue appeared on the right of the object, and yet instructed a left response; Olson, 2003). However, this response pattern occurred in SEF neurons previously trained to select targets using precisely an object-centered rule. Importantly, as discussed by Olson (2003), SEF neurons only show weak object-centered signals before training.

This training-dependence suggests that rather than responding to object-based spatial locations in a bottom-up manner (via object-centered *spatial selectivity*), such putatively object-centered neurons require extensive *training*, i.e., respond most likely to top-down signals. This could suggest *rule-based decision making signals* from other (perhaps dorsolateral prefrontal) regions, rather than *spatial perception* in an object-centered spatial reference frame. A testable prediction is that DLPFC activity should precede SEF activity on such tasks. In humans, a testable fMRI prediction would be that the effective connectivity between e.g., DLPFC and SEF should increase when the rule needs applied.

This interpretation of a *superimposition* of a rule onto SEF neuronal activity is also consistent with the fact that SEF neurons showed a modulation by egocentric saccade directions, i.e., a right-object selective SEF neuron still showed some preference for physically (egocentric) rightward saccades even if they fell on the (non-preferred) left end of the object (Olson, 2003).

Other studies have investigated object-centered representations in posterior parietal areas (for review, see Chafee and Crowe, 2012). Crowe et al. (2004) recorded from inferior parietal area 7a while monkeys were shown visual stimuli depicting octogonal mazes (viewed from the top), with a straight main path extending from the center box out (Figure 4C). In exit mazes, the main path exited to the perimeter, whereas in no-exit mazes, the main path ended in a dead end inside the maze. Monkeys mentally solved mazes to determine whether each maze had an exit path or not, without moving their eyes from the fixation point located at the center of each maze. While mentally solving the maze task, one quarter of neurons in parietal area 7a exhibited spatial tuning for maze path directions.

Interestingly, and consistent with the top-down hypothesis of object-centered processing, neuronal tuning for maze path direction only emerged after training (Crowe et al., 2004). In other words, naive animals that viewed the same maze stimuli without solving them did not show tuning to path direction. This argues against an existing, object-centered spatial representation, i.e., an “allocentric lens” through which spatial relationships in the world are viewed. If object-based spatial relationships did exist, these neurons should have represented them in a “bottom-up” manner just like retinocentric or egocentric spatial relationships are represented, which do not require task training. A neuron that has a preference for a certain object-centered spatial relationship (e.g., maze path exiting to the right of the maze) should exhibit such an object-centered preference whenever the monkey is looking at such a stimulus. It is possible that allocentric spatial tuning takes longer to develop with more complex visual stimuli, where multiple object-centered spatial relationships could be represented. Such training dependence, however, is also observed for simple bar stimuli, as reported by Olson (2003).

As in SEF, object-centered parietal area 7a neurons had a preference for contralateral path directions. In other words, neurons located in the left hemisphere preferred maze exits to the egocentric right. However, preferred maze path directions (e.g., up and to the right) were largely independent of receptive field (RF) locations as mapped with spot stimuli (Crowe et al., 2004). Spatial tuning for path direction in the maze task was also not systematically related to saccade direction tuning as mapped in an oculomotor control task. While this dissociation between the RFs mapped using control tasks and maze path direction would seem to suggest an independence of egocentric variables, it is also possible that individual neurons' RFs obtained with the visually more complex maze object shift dynamically with more complex tasks. The fact that the maze task needs solved mentally (without moving the eyes) would suggest that some mental remapping of information across receptive fields is necessary. I.e., neurons might dynamically and predictively represent the information expected to fall in their RFs if the eyes were moved. Thus, the classically defined RF location as mapped by spot light stimuli would seem less relevant than finding out what kind of remapping might be happening during mental solving of the maze task. Remapping of information even prior to saccades has been demonstrated in neighboring area LIP (Colby, 1998).

In fact, a subsequent study of the maze task (Crowe et al., 2005) studied the neuronal population dynamics during maze task solving. Crowe et al. (2005) found that following presentation of the maze, the population vector (the direction signaled by the majority of cells) in parietal area 7a began to grow in the direction of the exit path. In trials in which maze paths had a right-angle turn, the population vector rotated in the direction of the turn, however, not 90°, but 45°.

In other words, imagine a triangle corner centered at the fovea, with one triangle side extending vertically up from the fixation point; from the top of the vertical side, another side extends to the right, forming a right angle with the vertical line. If you were to move your eyes up one side of the triangle and then turn 90° right, the hypotenuse is 45° relative to the vertical meridian from your initial fixation point. In object coordinates, the configuration of the path toward the exit is first up, then 90° to the right. However, the populations of cells that became active were first cells preferring up, then cells preferring 45° to the right.

This is the vector angle one would expect if the vector origin were anchored to the *fovea* (initial fixation point), with the tip of the vector signaling the maze exit from the foveal origin to 45° up and to the right, as suggested by the authors (Crowe et al., 2005; Chafee and Crowe, 2012). This suggests the maze problem was solved from an egocentric, specifically retinocentric, perspective, and is less consistent with an object-centered representation, at least at the population level.

Another approach to studying object-centered spatial representations is to use a visual “object construction task” (Figures 4D,E), in which presentation of a model object consisting of a configuration of elements is followed by a test object in which one element is missing (Chafee et al., 2005, 2007). For instance, an inverted T-like structure consisting of Tetris-like blocks arranged vertically and horizontally was followed by a test structure where one block was missing left or right of the vertical object axis. Monkeys were trained to then “complete” the test object by choosing between two elements, one of which was on the correct side of the missing element location. Once the element was chosen, it was attached to the test object at the appropriate location.

By presenting either the test object or the model object at different retinal locations, Chafee et al. (2005, 2007) could investigate whether neurons in area 7a are sensitive to the object-referenced location of the missing element (e.g., top right of the object) regardless of the egocentric (retinal) location of the element. Chafee and colleagues found two populations of neurons in area 7a. One population coded the missing element in viewer-referenced (egocentric) coordinates, whereas a partially overlapping population encoded the missing element in object-referenced coordinates, signaling the missing piece both when the test object appeared left and right of the fixation cross. Object-centered neurons showed object-centered responses both when the whole shape (model) was presented, and when the test object (with a missing piece) was shown.

Several neurons indicated a joint viewer- and object-referenced influence, responding more strongly when both the element and the object were on the preferred side (for instance, both on the egocentric left and object-referenced left).

As in Olson (2003), this task (Chafee et al., 2005, 2007) allows either the model or the test object to be mentally translated to the ego-center (the fovea), where left or right on the object becomes a simple egocentric decision. The putative object-referenced population could thus be remapping locations in an ego-relative way. Consistent with the ego-relative interpretation, and similar to Olson (2003) and Crowe et al. (2004, 2005), object-referenced neurons preferred contralateral “missing elements” (relative to the object).

Moreover, the data suggest that this process is rule-dependent. In some trials, model objects contained two elements, one left and one right on the object, either of which could be removed in the test stage (Figure 4E). During the delay between model and test object presentation, the monkey could thus not know which of the elements would be removed for such ambiguous model objects. Interestingly, in contrast to trials where the element that would be removed was obvious during the model stage, there were no object-centered responses during presentation of the indeterminate model object, with object-centered responses only emerging after the test object revealed which element was missing.

Why would an object-centered neuron not signal “left on the object” regardless of which element (left or right) would end up being removed? It could be argued that if these neurons were encoding spatial locations relative to objects, then neurons selective for “left of the object” should have signaled the object-relative location of the element during the indeterminate model presentation as well as during test object (missing-piece-object) presentation. The fact that neurons “waited” until the missing element was revealed during the test object phase suggests that such neurons might encode *rules*, not *spatial relations*: at the moment of the ambiguous model, neurons could not yet apply any rule, since either of the two elements could be removed; the rule to be applied only emerged in the test stage. This suggests that these neurons do not have a true object-centered *spatial preference* such as “left on the object.” Rather, they encode the rule “detect if a certain ego-relative element is missing.” Thus, a coding of relative retinocentric position, rather than object-centered spatial reference frames, cannot be ruled out.

If neurons in inferior parietal area 7a are involved in mentally re-centering a peripherally-displayed visual stimulus such that it lines up with the fovea or ego center, one would expect object-centered responses that signal “object left” or “object right” regardless of retinal position to be somewhat delayed compared to simple egocentric responses. In fact, this is exactly what was found by Crowe et al. (2008). Information in retina-centered coordinates emerged first, and was followed by neural signals coding object-relative positions. The strength of egocentric and object-centered signals was correlated, and object-centered responses could be predicted from retina-centered responses, but not vice versa (Crowe et al., 2008). Thus, each location on an object is presumably first represented retinocentrically, e.g., for an object in the left visual field, the left edge of the object is represented as “further left” than the right edge of the object, which is represented as “left but closer to the midline.” These retinocentric coordinates are subsequently transformed into “object-left” and “object-right.” This is consistent with a mental shifting of the object to the ego-center, at which point the remapped (mentally shifted) “left” and “right” in object-based coordinates match the egocentric left and right.

Note that this suggests that allocentric reference frames are derived from egocentric reference frames, and are thus not at the same level in the computational hierarchy, i.e., object-based locations are not a primitive parameter in allocentric spatial processing in the same way egocentric locations constitute a primitive in egocentric spatial processing (Klatzky, 1998). This suggests that regardless of what level (which layers, or projections between layers in a multi-layer network) egocentric and allocentric computations take place at, the egocentric coordinates need computed first before being fed into a network that can construct object-based representations. The feasibility of transforming egocentric representations into object-directed responses using a basis function network that lacks explicit object-centered representations and whose neurons have retinotopic response fields, has been demonstrated by Deneve and Pouget (2003). Object-referenced actions emerge as mappings between the relative and absolute retinal locations of an object and particular motor commands—at no point in the network do such cells, or does the network, create an explicit object-centered spatial representation.

Even if a bottom-up transformation of egocentric to object-centered coordinates is possible, the rule-like behavior of some of these parietal neurons, and extensive training required to exhibit object-centered responses, however, also suggest a top-down modulatory signal. As with area SEF, this leads to the prediction of an earlier prefrontal than posterior parietal response. This prediction has in fact been tested. A recent study using simultaneous recordings in macaque prefrontal and posterior parietal cortex showed that rule-based spatial categorization signals are stronger and emerge earlier in dorsolateral prefrontal cortex than in area 7a (Goodwin et al., 2012). Monkeys were trained to categorize dots as either “left” or “right,” or “above,” or “below” a boundary in response to a rule cue. Thus, the same dot location could be classified as left or right, or as above or below, depending on the rule cue. Both parietal and prefrontal neurons represented spatial categories according to the rule, but with earlier and stronger rule-dependent modulation of category signals in prefrontal cortex, suggesting executive control over spatial processing.

This suggests the possibility that a number of object-relative responses found in area 7a and SEF are likely rule-dependent spatial responses, rather than *spatial perception* or *representations of spatial relations between objects* in a bottom-up manner, and explains why these object-relative responses (which likely depend on mental transformations of ego-centered responses) require a lot of training.

Finally, it should also be noted that, in contrast to object-based modulations of eye movement planning in SEF (Olson, 2003), posterior parietal area LIP, which is also involved in eye movement planning, did not show object-centered coding of saccade targets (Sabes et al., 2002). It is possible that this is due to the fact that the stimuli used by Sabes et al. involved the rotation of an irregular, asymmetric shape, in contrast to stimuli that can be mentally translated left or right to match the ego center. Another prediction therefore is that object-centered effects might disappear if rotations of more complex, asymmetric objects were employed, which make the mental transformation back to egocentric coordinates more difficult.

Outside parietal and frontal areas, medial superior temporal (MST) neurons have been reported to signal target motion independent of eye or head movements, possibly in a world-centered reference frame (Ilg et al., 2004). However, Sereno and Sereno (1991) have shown that position-independent, MST-like motion selectivity responses can develop in third-layer units of a feedforward network despite position-dependent direction selectivity within their receptive fields.

Chafee and Crowe (2012) distinguish between first-order (e.g., sensorimotor signals tightly coupled to stimuli or movements, in an egocentric frame of reference), second-order (signals are still dependent on e.g., egocentric position and movement parameters, but can be modulated by cognitive factors, such as attention, working memory, delayed planning), and third-order (complete sensorimotor independence both temporally and spatially) signals. The neurophysiology evidence on object-based spatial representations reviewed here is consistent with a highly abstract, cognitive signal. While it is debatable to what extent this abstract signal is independent of ego-relative parameters, it seems clear that these are high-level, cognitive signals that are likely “trained into the brain” (Chafee and Crowe, 2012).

## FMRI studies: brain activations for egocentric vs. allocentric tasks

While single-unit recordings are restricted to small numbers of brain regions, can neuroimaging reveal additional brain networks subserving allocentric spatial representations? Numerous fMRI studies have attempted to identify the neural substrates of allocentric and egocentric spatial processing (for reviews, see Galati et al., 2010; Boccia et al., 2014).

Despite the wide variety of tasks (and definitions) employed to probe allocentric spatial cognition, most studies fall into three broad categories: (1) spatial judgment tasks, e.g., tasks requiring subjects to report left/right locations relative to egocentric or object-centered coordinates (e.g., Galati et al., 2000; Neggers et al., 2006, similar to Figure 4B); or requiring spatial proximity or alignment judgments between two objects or objects and the ego (e.g., Saj et al., 2014); (2) spatial navigation tasks (virtual, imagined, or remembered; e.g., Committeri et al., 2004; Zhang and Ekstrom, 2013); and (3) allocentrically-guided movements, e.g., pointing or reaching to spatial targets relative to another object vs. relative to the ego (e.g., Thaler and Goodale, 2011; Chen et al., 2014).

In general, both egocentric and allocentric tasks have been reported to activate overlapping parieto-frontal networks, with generally greater egocentric than allocentric activations in superior parietal and superior frontal cortex, especially in the right hemisphere (Galati et al., 2000, 2010; Committeri et al., 2004; Neggers et al., 2006; Zhang and Ekstrom, 2013; Chen et al., 2014; Saj et al., 2014). Additional foci of greater egocentric than allocentric activation have been reported in superior or middle temporal gyrus (Neggers et al., 2006).

Note that despite evidence of overlapping parieto-frontal activations for both allocentric and egocentric tasks, no object-centered topographic maps have been found in parieto-frontal areas, across multiple attempts (Sereno et al., 2009), in contrast to well-established retinotopic or face-centered maps in parietal and prefrontal cortex (Hagler et al., 2007; Filimon, 2010; Sereno and Huang, 2014).

Allocentric tasks induce greater fMRI activations than egocentric tasks in temporal lobe structures and occipital regions, including the lingual gyrus (Galati et al., 2000; Committeri et al., 2004; Neggers et al., 2006; Chen et al., 2014); inferior temporal gyrus (Committeri et al., 2004; Zaehle et al., 2007; Saj et al., 2014); and hippocampus (Galati et al., 2000; Zaehle et al., 2007). Other fMRI studies have reported increased functional connectivity between the hippocampus, the superior parietal cortex, and precuneus in allocentric tasks (Zhang and Ekstrom, 2013). Thus, despite the overlap between egocentric and allocentric task activations, allocentric tasks rely more on ventral occipito-temporal networks, whereas egocentric tasks activate parieto-frontal networks more strongly (for an exception to the latter pattern, see Thaler and Goodale (2011) as well as Zaehle et al., 2007).

While this pattern is consistent with the idea of functionally and partially anatomically separate neural processes underlying allocentric and egocentric spatial cognition, here I examine whether different activation patterns (and ventral visual stream activations in particular) provide evidence for a separate allocentric *spatial reference frame*. I will argue that the different patterns of activation are task- and strategy-dependent, where the egocentric spatial frame is relied upon to varying degrees in combination with *non-spatial* object-recognition processes.

Regarding spatial judgment tasks, at first glance, the greater parieto-frontal activation for egocentric tasks reported by most studies appears puzzling. If allocentric processing involves mentally shifting or rotating objects to the egocentric midline, such that an object's left and right are concordant with the egocentric left and right, wouldn't this imply greater activation for allocentric than egocentric tasks, at least in posterior parietal cortex, due to allocentric tasks in fact relying on additional ego-relative processing?

In fact, such a pattern of greater parietal activation for allocentric tasks has been reported, and appears to depend on the nature of the task. Zaehle et al. (2007) for instance, used *verbal descriptions* of spatial relations instead of actual visual images. In the allocentric condition, subjects listened to descriptions of the location of geometric shapes (triangles, circles, squares) relative to each other (e.g., shape A was to the left of shape B, B was above C, shape C was to the right of D). Subjects were then asked to infer the spatial relationship between two shapes whose spatial relationship to each other had not been described, but could be inferred from the other objects (e.g., where D was relative to A). In the egocentric condition, spatial locations of objects were described relative to the body as well as relative to other objects (e.g., shape A is to your right; shape B is to the right of shape A), but subjects had to infer the spatial relationship of a target object relative to themselves (e.g., whether shape B was to their right).

Zaehle et al. (2007) found that, although both egocentric and allocentric conditions activated parieto-frontal regions, inferior temporal gyrus, and occipital areas, the allocentric condition led to greater activation than the egocentric condition in the right superior and inferior parietal lobule, the right superior and inferior frontal gyrus, the ventrolateral occipito-temporal cortex (inferior temporal gyrus), and the hippocampus.

These results are consistent with mental imagery of the different visual shapes. However, notice that here, *both* the egocentric *and* the allocentric conditions invoke mental imagery. The greater right parietal activations for the allocentric condition could be due to the additional effort of translating object-relative spatial locations of each object into egocentric coordinates, whereas in the egocentric condition, this relationship is already described. The allocentric task can be solved equally by keeping track of each object's location relative to oneself, and comparing the egocentric location of shape A and that of shape D. As argued in previous sections, it is possible to solve this type of problem in purely egocentric terms.

Why then have other studies reported the reverse pattern of greater parieto-frontal activations for egocentric than allocentric conditions? One possible reason is that in contrast to Zaehle et al.'s study, where *both* the egocentric and the allocentric conditions required mental imagery, most other fMRI studies use visual stimuli (e.g., Galati et al., 2000; Neggers et al., 2006). Spatial judgments based on actual egocentric visual stimulation may lead to stronger activations than spatial judgments based on imagined object translations back to the ego center in allocentric conditions.

For instance, Galati et al. (2000) and Neggers et al. (2006) both used a horizontal bar intersected by vertical lines at various positions relative to the bar midpoint. The horizontal bar was also displayed at various horizontal positions relative to the ego-center. In the allocentric condition, subjects had to report whether the vertical line was left or right of the horizontal bar midpoint, regardless of its egocentric position. In the egocentric condition, subjects reported whether the vertical line was to the left or right of their body midline. Both studies found stronger right posterior parietal activations for the egocentric condition compared to the allocentric task. Moreover, allocentric activations were much weaker overall, with neither study reporting significantly greater allocentric than egocentric activations. In Galati et al. (2000), there was a trend for greater medial occipital and hippocampal activation in the allocentric compared to egocentric condition, which however did not reach statistical significance. Both of these studies are consistent with more robust activation of posterior parietal cortex when the egocentric spatial location is presented visually rather than mentally imagined. Alternatively, it is possible that establishing the egocentric “body midline” may require greater effort due to less precise proprioceptive mechanisms, compared to estimating simple retinal distances in the allocentric condition, thus leading to greater activation in the egocentric condition. Similar results were also obtained by Saj et al. (2014), who used vertical alignment judgments between two shapes vs. between one shape and the egocentric midline, as allocentric and egocentric tasks, respectively. This task can also be solved in purely egocentric terms, by calculating the retinocentric vector from the fovea to each shape. If one vector is longer than the other, clearly the two shapes are not aligned with each other. Similar to other studies, Saj et al. also obtained stronger right posterior parietal activations for the egocentric compared to the allocentric task, and greater allocentric than egocentric activation in left inferior temporal cortex.

Other fMRI studies have compared egocentric vs. allocentric tasks in spatial navigation or more complex virtual environments. Committeri et al. (2004) used snapshots of a virtual environment taken from different points of view, representing a central square with a fountain and a three-winged palace surrounding it. Inside the courtyard, two target objects and a reference object were displayed at different spatial distances to each other, to the central wing of the palace, and to the subject. Subjects had to decide which of the two target objects was closer to them (viewer-centered condition), which was closer to the reference object (object-centered condition), and which was closest to the central wing of the palace (landmark-centered condition). Note that each of these conditions is equally solvable in egocentric terms: during training, subjects learn view-specific layouts of the environment, together with where the central wing is relative to them, in each scene. Hence deciding which target object is closer to the central wing of the palace (landmark condition) could be solved by first establishing whether the central wing is on the egocentric right or left, and which of the two target objects is more right or left, in egocentric terms. Deciding which of two target objects is closer to a reference object (object-centered condition) likewise involves estimating which retinal distance between two points (target 1 and reference object, or target 2 and reference object) is shorter, together with depth and size cues of objects that are nearer or farther. In contrast to the other conditions, the landmark condition additionally requires retrieval of different (viewpoint-dependent) scene views from memory. All three conditions activated posterior parietal cortex. The main differences consisted of a bilateral ventro-lateral occipito-temporal activation (inferior temporal gyrus) present only in the object-centered condition, and medial occipito-temporal (fusiform, lingual gyrus and parahippocampal cortex) activations in the landmark condition (which relied on scene recognition).

Thus, the ventral visual stream activations are consistent with representations of visual distances (similar to Saj et al., 2014, where the retinal distance between two objects had to be estimated in the allocentric task). This is also consistent with patient D.F.'s deficits discussed in the Neuropsychology Section above. The medial occipito-temporal activations are consistent with view-dependent scene recognition. While *allocentric spatial frames independent* of the viewer's perspective could be postulated, the alternative egocentric explanations are at least as likely.

Also supporting the interpretation that directly perceived egocentric coordinates activate spatial networks more than ego-relative mental transformations, Zhang and Ekstrom (2013) found that a simple control condition of just navigating to a visible target led to as much, if not more, activation as various imagined mental transformations necessary for navigating from one landmark to the next, in retrosplenial cortex, precuneus, parahippocampus, and superior parietal cortex. This is consistent with Bennett's (1996) hypothesis that animals navigate most efficiently based on recognized landmarks by moving toward them, rather than by using cognitive maps of ego-independent allocentric spatial relationships between landmarks.

Moreover, Huang and Sereno (2013) recently showed that the mental navigation network, which includes retrosplenial cortex, posterior parietal, premotor, precuneus, parahippocampal, and occipital regions, largely overlaps with retinotopic, and hence egocentric, maps. In fact, they suggest that this bottom-up retinotopic organization helps encode scene and location information in an *eye-centered* reference frame for use in top-down, mentally simulated navigation.

The greater reliance on ventral visual or temporal lobe activations in some allocentric tasks could thus be interpreted as tasks that place greater memory or mental navigation demands—but nevertheless from an egocentric perspective, e.g., remembering sequences of landmarks from an egocentric perspective.

Finally, other fMRI studies have investigated the use of allocentric and egocentric frames of reference in the context of planning and executing movements toward remembered or remapped targets (Thaler and Goodale, 2011; Chen et al., 2014). Although a shift from dorsal to ventral visual regions has been proposed for immediate vs. delayed movements, respectively, both dorsal and ventral visual stream areas are re-activated at the time of delayed movements, with greater reliance on ventral areas in the case of delayed grasping compared to pointing, presumably because detailed visual information about object size and shape needs re-activated (Singhal et al., 2013).

In a study by Chen et al. (2014) differently colored horizontal dots indicated the fixation point, target, and allocentric landmarks. The fixation dot appeared first, followed by a target together with a landmark cue, at various horizontal eccentricities. The target and landmark then disappeared, and the fixation point was shifted to the center. Following a 12 s delay, the landmark reappeared either at the same or different location and an auditory reach instruction was given. In the egocentric conditions, subjects either reached to the remembered egocentric location of the target (pro-reach), or to the opposite location of the egocentric target (anti-reach). In the allocentric condition, subjects reached to the remembered location of the target relative to the allocentric landmark (i.e., if the landmark had shifted, the implied reach target shifted with it). In control trials, subjects reported the color of the target.

During the delay, the exact location of the future reach target could not be predicted, since the allocentric landmark could re-appear at novel locations relative to the fixation point. Similarly, the egocentric target location could be revealed as either the remembered location or the opposite location (although in principle subjects could be maintaining two simultaneous egocentric target locations in working memory). However, since targets and landmarks consisted of differently colored dots subjects could presumably rehearse the target-landmark configuration as a unit, akin to an object configuration, in the allocentric condition (e.g., a red and blue dot for target left, landmark right, respectively). As argued previously, this configuration remains dependent on how this arrangement appeared from the ego perspective (see Figure 1 and Section Theoretical Considerations).

As expected, Chen et al. (2014) found that during the delay, both egocentric and allocentric target encoding activated parietal and premotor areas. However, egocentric encoding of target position activated the posterior parietal lobe and PMd (dorsal premotor cortex) more strongly than the allocentric target encoding condition. Conversely, during the delay, the allocentric condition led to greater activation in the lingual gyrus, cuneus, and calcarine, i.e., all visual areas. Note that this is consistent with a *spatial* encoding in the egocentric condition, but a more *visual configuration*, similar to *object processing*, in the allocentric condition.

Thus there obviously *is* an effect of “allocentric” cues—however, it is debatable whether this should be interpreted as an *allocentric spatial reference frame* effect rather than a ventral visual stream, *object configuration* or *object processing* effect, where multiple visual stimuli are treated as a unit (c.f. Logothetis, 2000).

Effects of “target left of the allocentric landmark” vs. “target right of the allocentric landmark” during the delay were also constrained to the ventral visual pathway, namely the inferior temporal gyrus and inferior occipital gyrus (Chen et al., 2014). This is also consistent with a retinotopic representation of an object, with egocentrically more left vs. more right locations activating object processing areas that contain retinotopic visual maps (Huang and Sereno, 2013).

Although Thaler and Goodale (2011) found the opposite pattern (allo > ego in parieto-frontal circuits) for cursor movements to allocentrically-defined targets, the delay and movement planning phases were not separated, and allocentric targets could have been immediately converted to egocentric coordinates from the beginning.

In summary, fMRI studies have generally shown a pattern of overlapping activations in parieto-frontal regions for allocentric and egocentric tasks, which can presumably be explained by the common translation of both “egocentric” and “allocentric” targets into ego-relative coordinates. Additionally, regions specific to allocentric spatial judgment tasks overlap with ventral visual areas involved in object and object configuration processing. Allocentric tasks that involve mental navigation between different landmarks presumably involve additional mental transformations of the ego into different imagined orientations, hence activating hippocampal and related (e.g., retrosplenial) regions that encode or store multiple view-dependent scene representations during navigation. Although the activation patterns for egocentric and allocentric tasks are partly distinct, they do not unequivocally support the existence of *allocentric spatial reference frames*, and could thus be reinterpreted using egocentric reference frames alone.

## Conclusions and predictions

The evidence reviewed here, spanning behavioral, neuronal, neuropsychological, and neuroimaging studies, suggests that allocentric spatial representations may not be independent of egocentric coordinates, whether for navigation, spatial perception, or target-directed movements. Both empirical evidence and theoretical considerations suggest that spatial mechanisms relying only on egocentric reference frames cannot be ruled out. Egocentric explanations for allocentric effects have been proposed before (e.g., Bennett, 1996; Mozer, 1999; Driver and Pouget, 2000; Wang and Spelke, 2002; Deneve and Pouget, 2003; Rorden et al., 2012). This review has attempted to unify a wide variety of findings from multiple fields of investigation, and to show how egocentric mechanisms could account for allocentric task effects in multiple domains.

Not all spatial judgments rely on the *immediate* (present) egocentric perspective, but can involve mental transformations such as imagined rotations and translations of the ego or of objects (*ego-relative* coordinates). Moreover, abstract rules *can* be built upon ego-relative spatial representations, as discussed in the Neurophysiology Section. Similarly, for recognition of landmarks to be possible from multiple viewpoints, viewpoint-invariance needs to be established.

This suggests a hierarchy of representations, including parieto-frontal egocentric spatial representations and ego-relative remapping, landmark and object recognition in the ventral visual stream, path integration in hippocampal and related structures, and categorical rule representation involving prefrontal networks. While these networks undoubtedly work together to solve the many complex spatial tasks that animals face, the key argument made here is that bottom-up *spatial* representations are fundamentally ego-dependent.

What criteria could be used to evaluate whether an allocentric spatial reference frame is used? Some possible criteria include:

1. Controlling for egocentric confounds, by varying the spatial location of objects not only relative to the eyes and head, for example, but also the body or any other possible egocentric reference frame (hand, foot, etc.). An object-centered spatial reference frame would be suggested if a cell's response does not depend on the object's spatial relationship to any body part.
2. To rule out learning of rule-based categorization, allocentric response fields tied to an object or part of an object should be present without extensive training, similar to egocentric receptive fields.
3. To identify whether cells encode configurations of object features holistically or conversely relative to each other in allocentric coordinates, a cell representing feature A relative to feature B in the object should continue to signal that spatial relationship if different parts of the object are removed. Similarly, if in a scene object A is represented relative to object B, moving object B should shift the allocentric response field tied to that object, such that a cell should respond to object A at the new, updated allocentric location, even if other objects in the scene have not moved.

Other specific testable predictions include:

1. At the behavioral level:
  - the improved spatial localization accuracy when presenting spatial targets relative to landmarks should disappear if the allocentric landmark is an irregular shape that is *rotated* between initial and post-delay presentations. Conversely, if the target is encoded in an object-centered reference frame, rotation of the landmark should have no effect on accuracies (or on reaction times), since the allocentric relationship should be independent of the egocentric perspective.
2. At the neuropsychological level:
  - hemineglect patients would be expected to show no object-based neglect for novel objects that are radially symmetric or which lack an intrinsic longitudinal axis that could be mentally rotated upright to match an egocentric, viewpoint-dependent representation of such an object. Instead, the egocentrically-defined contralesional half of such unfamiliar objects would be expected to be ignored in any orientation. The lack of a canonical upright orientation for such objects predicts that mental rotations should not take place for these objects.
  - object-based neglect will vary as a function of encoding vs. retrieval, and familiarity with an object. In other words, object-centered neglect should appear for novel objects experienced in a particular orientation over and over again, as a view-dependent mental representation becomes established over time.
3. At the neural and neuroimaging level:
  - brain activity for object-based spatial decisions should be slower than for egocentric spatial decisions (note that behavioral reaction times may not be sensitive enough to detect such temporal delays). EEG or MEG, or event-related fMRI and effective connectivity, could establish the time courses of different brain networks during “allocentric” and “egocentric” tasks. Egocentric decisions should show an earlier temporal profile compared to allocentric decisions, at least in parieto-frontal networks associated with space perception. Rule-based spatial decision making should activate prefrontal decision making regions such as DLPFC (Filimon et al., 2013) earlier than parieto-frontal spatial networks.
  - the fMRI literature suggests that parietal (or parieto-frontal) activations should generally be stronger for *seen*, rather than *imagined*, spatial relations. Stronger activations for visual observation than imagery, or for visible compared to invisible reaching, have indeed been reported in the posterior intraparietal sulcus and high-level visual areas (Filimon et al., 2007, 2009, 2015).
    However, the more difficult the (allo-to-ego) mental transformation required for an allocentric stimulus (e.g., mental rotations, etc.), the stronger the activation should be.

The current review demonstrates several difficulties and challenges in teasing apart allocentric spatial reference frames, non-spatial mechanisms, and egocentric representations. The examples given here illustrate that it is possible to explain a wide variety of allocentric task effects using egocentric spatial reference frames. The interpretation offered here is of course only one possible interpretation, and it is certainly possible to refer to object recognition as “allocentric” if what is meant by that is the ability to categorize multiple viewpoints as the same object. However, this is not necessarily an agreed-upon definition. Future studies could test the specific predictions made by the egocentric account and control for alternative non-spatial explanations. A clear and consistent definition of the term allocentric will be a key step in this direction.

### Conflict of interest statement

The author declares that the research was conducted in the absence of any commercial or financial relationships that could be construed as a potential conflict of interest.
